# Supplementary material for: An intrinsic tumour eviction mechanism in Drosophila mediated by steroid hormone signalling
Source: Nat Commun. 2018 Aug 17;9:3293. doi: 10.1038/s41467-018-05794-1 (PMC6098038; doi:10.1038/s41467-018-05794-1)
Supplement: Supplementary file 1 — Supplementary Information [file 41467_2018_5794_MOESM1_ESM.pdf]

Supplementary Information

An intrinsic tumour eviction mechanism in Drosophila mediated by steroid hormone signalling

Jiang et al.

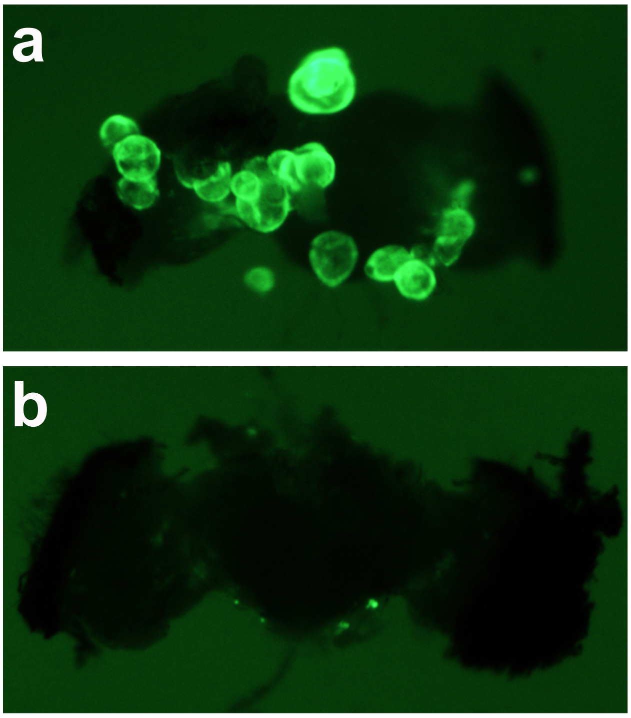

Supplementary Figure 1. **a**, GFP-marked *ph*^{505} cells form grape-like structures in the dissected head of one-day-old adult flies. **b**, Metamorphed *ph*^{505} cells in the head disappear almost completely after seven days.

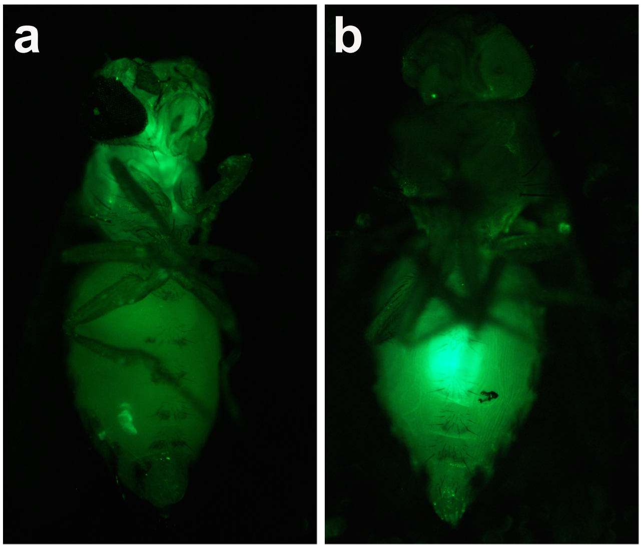

Supplementary Figure 2. **a**, An adult fly of the genotype *ph*^{505}*; UAS-RNAi-usp* at two weeks after eclosion, showing the formation of tumours all over the body. **b**, Formation of tumours in a wild-type host after transplantation of adult *ph*^{505}*; UAS-RNAi-usp* cells dissected from the head.

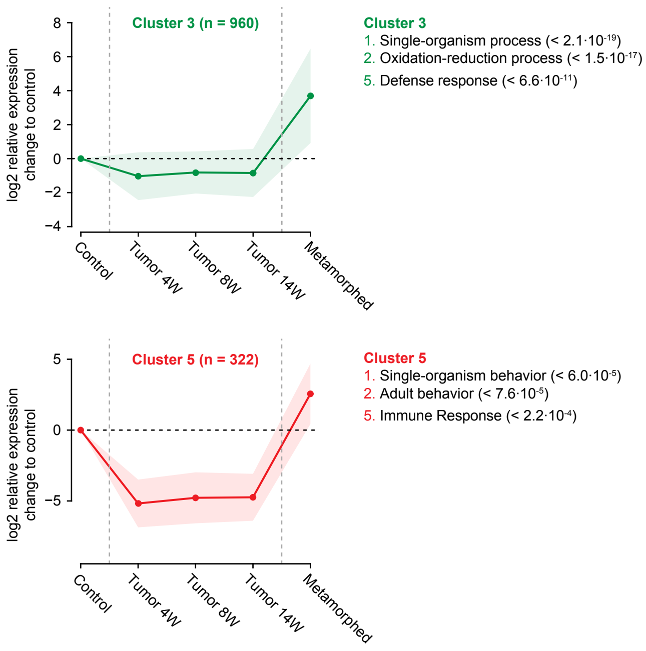

Supplementary Figure 3. Clustering analysis of significantly differentially expressed genes between control, *ph*^{505} tumours and non-tumorigenic metamorphed *ph*^{505} cells. All n genes of each cluster lie within the shaded area, whereas the line denotes their mean.

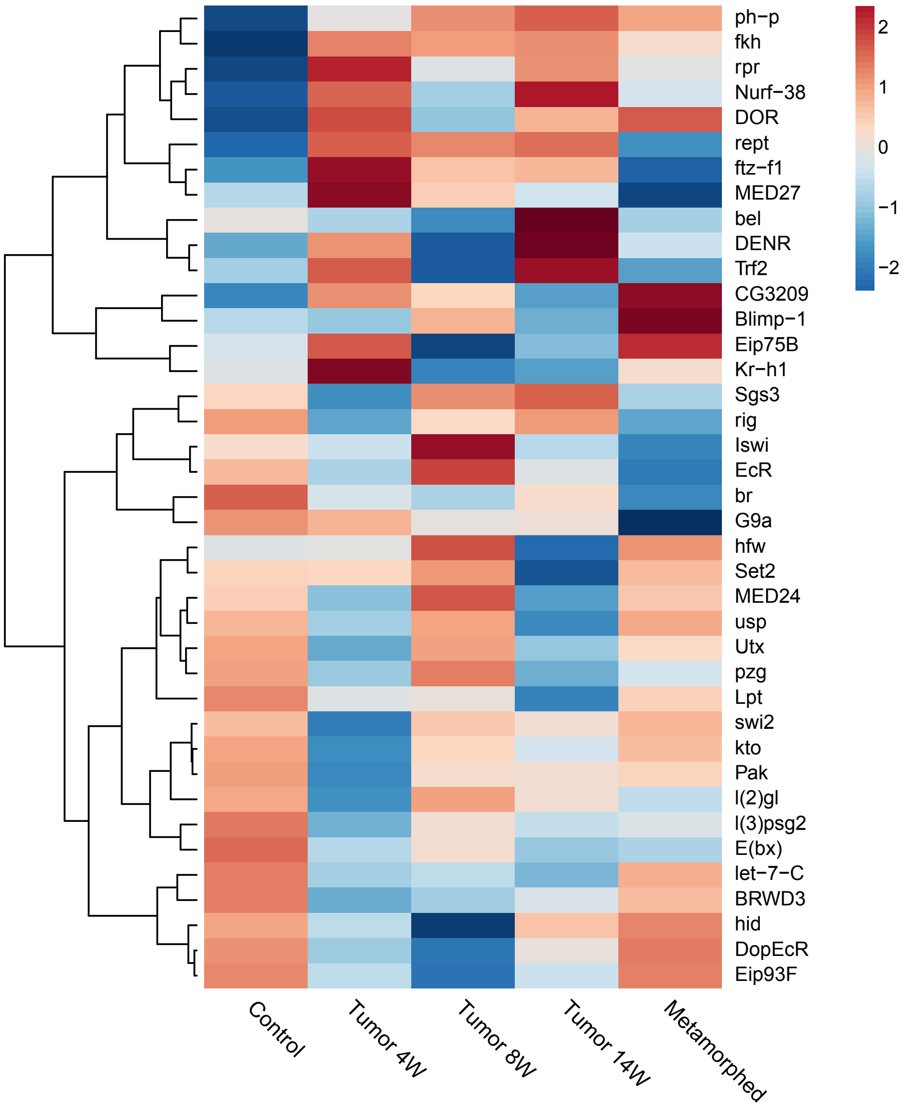

Supplementary Figure 4. Hierarchical clustering showing the expression level of all genes known to respond to ecdysone in control, tumour samples and metamorphed *ph*^{505}cells.

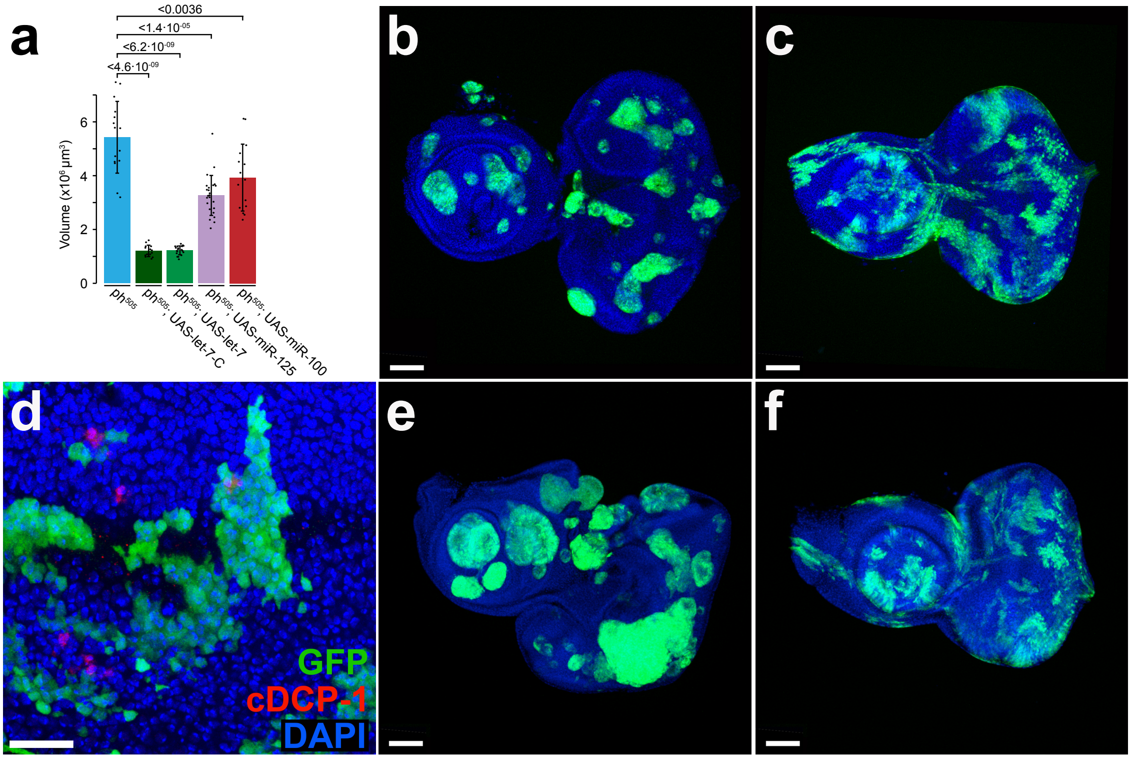

Supplementary Figure 5. **a**, Mean total volume of tumour clones in the eye-antennal discs of the different genotypes. n=15 (*ph*^{505}), 14 (*ph*^{505}*; UAS-let-7-C*), 19 (*ph*^{505}*; UAS-let-7*), 24 (*ph*^{505}*; UAS-miR-125*), and 15 (*ph*^{505}*; UAS-miR-100*), with error bars denoting the standard deviation. Significance was assessed using Welch's test. **b-f**, Representative confocal images of the eye-antennal discs at wandering third instar containing clones of *ph*^{505}; *UAS-let-7* (**b**), *F19A*; *UAS-let-7* (**c, d**), *ph*^{505}; *UAS-RNAi-chinmo* (**e**), and *F19A*; *UAS-RNAi-chinmo* (**f**). Note in **d**, most of the GFP-labelled cells over-expressing *let-7* do not show the expression of the apoptosis cell marker cDCP-1. Scale bars are 50 µm (**b, c, e, f**) and 20 µm (**d**).

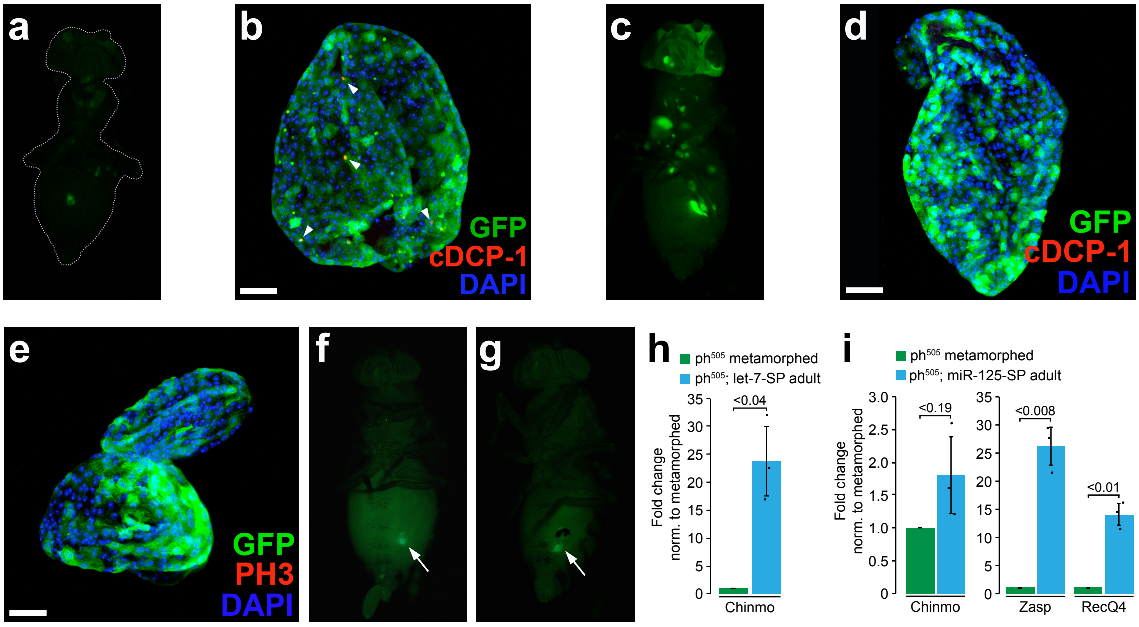

Supplementary Figure 6. **a**, A representative picture showing *ph*^{505}*; UAS-mir-100-SP* cells stopped growing and disappeared in a two-week-old adult fly (indicated by dashed line). **b**, Apoptosis cell marker cDCP-1 was expressed in the *ph*^{505}*; UAS-mir-100-SP* cells (arrowheads). **c**, A representative image showing *ph*^{505}*; UAS-mir-125-SP* cells were still visible in a two-week-old adult fly. **d**, Apoptosis cell marker cDCP-1 was not expressed in the *ph*^{505}*; UAS-mir-125-SP* cells. **e**, Mitosis cell marker PH3 was not expressed in the *ph*^{505}*; UAS-mir-125-SP* cells. **f**, **g**, Adult *ph*^{505}*; UAS-mir-125-SP* cells were transplanted into host flies (f, arrow), they did not give rise to neoplastic tumours but were still present after 4 weeks (g, arrow). **h**, Quantitative PCR analysis of the expression level of *chinmo* in *ph*^{505} metamorphed cells and in adult *ph*^{505}*; let-7-SP* cells. **i**, Quantitative PCR analysis of the expression level of *chinmo* and two candidate targets of *miR-125* in *ph*^{505} metamorphed cells and in adult *ph*^{505}*; UAS-mir-125-SP* cells. **h, i,** Bars denote the mean and standard deviation of three biological replicates (of three experiments conducted in total). P-values were calculated using the T-test. Scale bars are 50 µm in all panels.

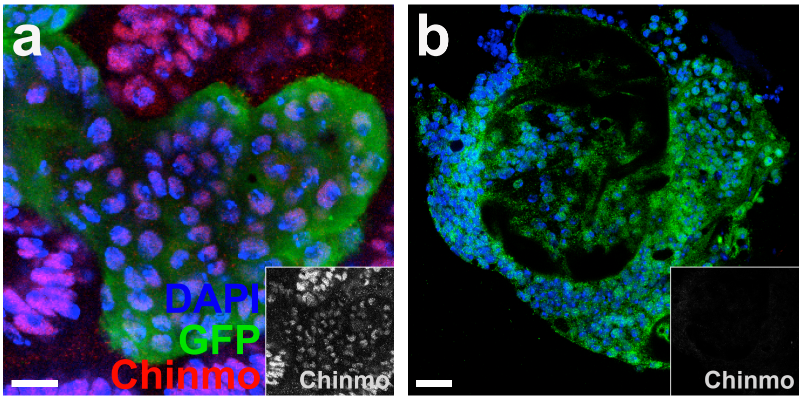

Supplementary Figure 7. Confocal images of immunostaining with Chinmo antibodies. At the third larval stage, Chinmo is expressed in the *ph*^{505} clones in the eye discs (**a**), scale bar is 10 µm. At 48 hours after pupal formation, the expression of Chinmo is undetectable in the *ph*^{505} cells (**b**), scale bar is 20 µm.
